# What is important for people with type 2 diabetes? A focus group study to identify relevant aspects for Patient-Reported Outcome Measures in diabetes care

**DOI:** 10.1371/journal.pone.0277424

**Published:** 2022-11-14

**Authors:** Nura Abdel-Rahman, Orly Manor, Liora Valinsky, Ofri Mosenzon, Ronit Calderon-Margalit, Sveta Roberman

**Affiliations:** 1 Braun School of Public Health, Hebrew University of Jerusalem Hadassah Medical School, Jerusalem, Israel; 2 Meuhedet Health Services, Tel Aviv, Israel; 3 Diabetes Unit, Department of Endocrinology and Metabolism, Hadassah Medical Center, Jerusalem, Israel; 4 Faculty of Medicine, Hebrew University of Jerusalem, Jerusalem, Israel; 5 Gordon Academic College of Education, Haifa, Israel; Chiang Mai University, THAILAND

## Abstract

**Background:**

Patient-Reported Outcome Measures (PROMs) aim to evaluate the quality of care based on the perspectives of patients rather than clinical indicators. Qualitative research is needed to identify these perspectives in people with type 2 diabetes.

**Objective:**

To identify, for the first time in Israel, aspects valuable for people with type 2 diabetes that can be relevant for PROMs in diabetes care.

**Methods:**

A qualitative study included three focus groups totalling 19 people with type 2 diabetes. Inclusion criteria were: (1)type 2 diabetes, (2)diabetes duration of at least six months, and (3)adults aged 45–80 years. Purposive sampling enabled recruitment of heterogeneous participants. Also, two experts’ panels with healthcare providers involved in diabetes care (n = 23) were conducted to provide triangulation of information (more testimony about what is valuable for people with type 2 diabetes). Discussions were recorded, transcribed and thematically analysed.

**Results:**

Four domains were deemed valuable for people with type 2 diabetes: (1)challenges of living with diabetes, including reduced physical function, healthy lifestyle struggles, sexual dysfunction, and financial burden, (2)mental health issues, including depression, distress, anxiety, frustration, and loneliness, (3)self-management ability, including management of lifestyle modifications and treatment, knowledge about the disease and treatment, and (4)patient-clinician relationships, including the devotion of clinicians, trust in clinicians and treatment, shared decision-making, and multidisciplinary care under one roof. Experts favour using PROMs in diabetes routine care and even acknowledged their necessity to improve the treatment process. However, only some of the domains raised by people with type 2 diabetes were identified by the experts.

**Conclusions:**

There are content gaps between perspectives of people with type 2 diabetes and their healthcare providers. PROMs are essential in addressing issues largely not addressed in routine diabetes care. We recommend that researchers and healthcare providers, who intend to utilize PROMs for diabetes care, consider the aforementioned domains.

## Introduction

Healthcare has become more patient-centred in recent decades, with the measurement of quality of care gaining increasing attention [1, 2]. Evaluating quality of care based on clinical indicators, while highly important, may not capture all relevant aspects of care and it is important to measure outcomes that matter most to patients [3]. A series of articles in the New England Journal of Medicine have called for a reform in measuring and improving healthcare, based on patients’ perspectives, rather than clinical indicators [1, 2, 4], leading to the emergence of Patient-Reported Outcome Measures (PROMs) [5].

Patient-Reported Outcomes are defined as ‘any report coming directly from patients, without interpretation by physicians or others, about how they function or feel in relation to a health condition and its therapy’ [5]. PROMs are usually accessed with two types of questionnaires: generic and disease-specific [6]. PROMs, increasingly used in procedures (e.g., hip or knee replacements) and in the field of oncology, are associated with improved symptoms management, enhanced psychological well-being, and longer survival [7, 8]. PROMs were developed to address the gap between patients’ perspectives and the healthcare providers’ perspectives. Thus, to truly capture aspects that are meaningful to patients, it is necessary to involve them in PROMs development. However, a scoping review showed that while in 74% of processes of PROMs development patients were somehow involved, only 11% of these processes had patients actually involved in the decision making of which outcomes to include [9].

Diabetes care aims to prevent complications and maintain people’s quality of life [10]. The American Diabetes Association (ADA) recommends routine monitoring of these aims [10, 11]. Since the 1990s, quality of life is recognized broadly as an essential health outcome in diabetes [12, 13]. Several review papers and multinational studies have addressed PROMs (including, but not limited to, quality of life) in diabetes [13–17], various tools were used to assess PROMs with a broad range of domains and heterogeneous content. Notably, perspectives of people with type 2 diabetes may well differ between countries, cultures, and healthcare systems. So far, there are no PROMs for diabetes that are widely used and it is unclear which aspects of PROMs should be measured in people with type 2 diabetes. Also, not all PROMs are informed by perspectives of people with type 2 diabetes with the risk of missing aspects valuable to them. Only a few studies have considered perspectives of people with type 2 diabetes to develop PROMs, some of them have built new tools and others have adapted previously validated tools and added newly developed items [18–21]. Therefore, there is still a need to identify perspectives of people with type 2 diabetes. Thus, we aimed to identify, for the first time in Israel, aspects valuable for people with type 2 diabetes that can be relevant for PROMs in diabetes care.

## Materials and methods

### Study design and setting

The current study is a focus group (FG) qualitative study. It is part of a larger study that conducted in the framework of the Israeli National Program for Quality Indicators in Community Healthcare (QICH), which aimed to incorporate PROMs for diabetes within the Israeli national quality indicator set for diabetes. The QICH quality indicators include clinical indicators (e.g., glycated hemoglobin-HbA1c testing and control) that were chosen and updated based on the best available evidence, as well as on national and international guidelines, with a consensus of representatives from professional organizations [22, 23].

### Study sample

To be invited to the FG, people had to be diagnosed with type 2 diabetes, and have the disease for at least six months. We included only adults aged 45–80 years old, as type 2 diabetes mostly occurs after the age of 45 years [24]. We did not include people with type 2 diabetes who were older than 80 years for two reasons–one is the increased number of comorbidities that makes it more difficult to discuss the isolated burden of diabetes, and the second is the relatively more relaxed guidelines among elderly patients in terms of managing diabetes. We excluded from the study individuals who could not participate in the discussions due to intellectual disabilities and those who declined to participate.

Purposive sampling (heterogeneous) was utilized to recruit people with diverse demographics (age, gender, and ethnicity) and diabetes duration based on electronic medical records. Eligible patients were contacted by phone, they received explanations about the study and were invited to participate. To ensure heterogeneous sample, participants were recruited from the outpatient clinics of ‘Hadassah Medical Center’ (one of the largest hospitals in Israel) and primary health clinics of the ‘Meuhedet Health Services’ (one of Israel’s four health plans that supply primary healthcare to all Israeli citizens).

Data saturation, which is the most common guiding principle in qualitative research [25, 26], was used to determine the adequacy of sample size. Thus FGs were continued till data was saturated and no new information emerged. This study included three FGs of 19 people with type 2 diabetes.

### Data collection

FG sessions were conducted between May 2017 and March 2018, in a private conference room in the hospital or at the health plan, and each lasted for 90 minutes. Prior to beginning the discussion, people with type 2 diabetes signed informed consent form and then completed an anonymous short demographic questionnaire. The FGs began with a brief description of the aim of this study and participants were informed that they had the right to leave the FG at any time.

A semi-structured open-ended topic guide (S1 File) was used to guide FG discussions. The topic guide was developed in line with the research questions and literature review (by N.A., O.M.1 and R.C.M). Before conducting the first FG, the topic guide was checked by an expert in FG discussion to ensure that the questions were clear, and changes were made as needed. The topic guide consists of seven questions asking participants to describe their experiences living with diabetes, opinions on diabetes care and how they characterized good diabetes quality care.

Two of the groups were conducted in Hebrew, and one conducted in Arabic, according to the participants’ spoken language. The majority of the population in Israel is Jewish (Hebrew speakers) with an Arab minority (Arabic speakers). The Jews and Arabs differ in their religion, culture and socio-demographic characteristics. We deliberately constructed a FG among Arabs to reflect the diversity of the ethnicity. All FGs discussion were led by the first author (N.A.) who is bilingual (Arabic and Hebrew) and notes were taken during FGs discussion by a research assistant (Arabic or Hebrew speaker). Interactions between participants provided meaningful insights and there was a sense of motivation and openness among participants in the discussions. The discussions were recorded with participants’ consent.

A professional transcription company transcribed (by an Arabic or Hebrew speaker according to the FG participants’ spoken language) the FG discussions from the digital recording to Microsoft Word files. A comparison between recordings and transcripts were done by the first author to ensure accuracy.

### Experts’ panels

To achieve a comprehensive understanding of the research questions we used triangulation of information [27], i.e. in addition to the perspectives of people with type 2 diabetes, we present in this article perspectives of 23 healthcare providers involved in diabetes care. The combination of patients’ and experts’ perspectives allows us to illuminate major experiences and challenges of diabetes care. The second aim of the experts’ panels was to learn whether they are in favor of using PROMs for diabetes, since their support is crucial for routine measurement. And the third aim was to compare between the patients’ and experts’ perspectives and to identify how they differ.

To attain a variety of opinions and a representative sample, healthcare providers were recruited based on their specialties: diabetes nurses, family physicians, diabetes physicians, social workers, and quality of care and PROMs experts.

Two experts’ panels were conducted, the first panel took place within a conference initiated by the Israeli Ministry of Health that aimed to promote PROMs in Israel and the second panel was conducted as a continuation of the first one. The experts’ panels were led by N.A., O.M.1 and R.C.M., and were conducted in Hebrew (according to the participants’ spoken language). The experts’ panels took place between May 2017 and March 2018 (concurrently with the FGs). The experts’ panels began with a brief introduction about PROMs, followed by asking participants to describe their experiences of diabetes care, perceptions of valuable aspects of diabetes care for people with type 2 diabetes and opinions regarding PROMs. The discussions were recorded with participants’ consent and later transcribed.

### Data analysis

The data were thematically analysed [27, 28] concurrently with data collection; each transcript was read several times, divided into meaning units (unitizing) and then meaning units within the same topic were categorized into the same domain. Domains were labelled based on the natural language of the participants (in-vivo). Data analysis were done independently by two of the researcher (N.A. and S.R.). Interpretations, domains and sub-domains were discussed with O.M.1 and R.C.M., differences were discussed and resolved. Analyses of the discussions of the third FG resulted in repetitive domains, i.e., data saturation had been reached.

### Research group

The research group consists of one PhD student (N.A.), two professors-supervisors (O.M.1 and R.C.M.) and three researchers (S.R., L.V. and O.M.2) with extensive experience in research in diabetes care and qualitative research. None of the participants in the experts’ panels was part of the research group. The research was part of the PhD thesis of the first author (N.A.). Prior to the current study, N.A. underwent training in leading qualitative research including FG discussion, data collecting and analysis.

### Ethical approval

This study was approved by the Helsinki Committee of ‘Hadassah Medical Center’ and ‘Meuhedet Health Services’. Written informed consent was obtained from all participants in the FGs.

## Results

People with type 2 diabetes participated in the FGs had a mean age of 65.1 years (range: 45.2–76.8). Participants were diverse (men:12 and women:7, Jews:14 and Arabs:5, born in Israel:12 and immigrants:7). All religious groups were represented, and all but three participants were married. The median time since diagnosis of diabetes was 14.5 years (range: 0.5–36 years), and 8 participants were receiving insulin treatment.

Participants in the experts’ panels (men:10 and women:13) had diverse professions (diabetes nurses: 3, family physicians: 5, diabetes physicians: 6, social workers: 2, and quality of care and PROMs experts: 7). All participants had expertise in the treatment of diabetes in the community.

### Valuable aspects for type 2 diabetes

The analyses revealed four domains that were deemed important for people with type 2 diabetes: (1) challenges of living with diabetes, (2) mental health issues, (3) self-management ability and (4) the patient-clinician relationship. The following describes these domains and their sub-domains and provides exemplifying quotes.

#### 1. Challenges of living with diabetes

This section introduces five main challenges emphasized in the groups: reduced physical function, healthy lifestyle struggles, hypoglycaemia, sexual dysfunction and financial burden due to diabetes.

**Physical function and fatigue** were raised frequently by people with type 2 diabetes (PWD). Diabetes reduced physical function, which was accompanied by fatigue. For example, a 45 year old man reported that after his diabetes diagnosis he was: ‘(…) *more tired in the afternoon’* (PWD, man, FG-3). Participants mentioned that feelings of fatigue affected their daily lives, limiting them in several respects, including performing home duties, engaging in physical activity and work productivity: ‘*Walking*, *functioning*, *home duties and dishwashing [is hard] because sometimes diabetes is very exhausting’* (PWD, woman, FG-2).

Additionally, a PWD noted: ‘*You are less productive at work (…)*. *There are things that are hard for me because of the fatigue*, *so I need workers to help*. *[Other*: *Do you relate it to diabetes*?*] Of course’* (PWD, man, FG-3).

Several participants voiced a desire for a treatment to eliminate fatigue: ‘*I want a medication or advice on how to behave to eliminate fatigue*, *at least’* (PWD, woman, FG-1). It is worthy to mention that some patients, mainly newly diagnosed individuals, wondered if fatigue was related to diabetes: ‘*I suffer from fatigue*, *but I don’t relate it to diabetes*. *Now I am hearing from everybody that it could be; I’m very tired*, *I cannot walk for a long time*, *I feel fatigued (*…*) Now when I hear from others*, *maybe it is related to diabetes*, *I don’t know’* (PWD, man, FG-3).

*Healthy lifestyle struggles*. Leading a healthy lifestyle, especially regarding diet and physical activity, emerged as essential challenges for people with diabetes, who reported that they wanted and tried to achieve a healthy lifestyle, but it was difficult to achieve. Regarding diet, people with diabetes noted that they faced many restrictions, stating that they were limited in their food options and that maintaining a healthy diet was difficult, particularly for those working outside the home.

*‘The diet is very difficult*, *it’s so hard*. *Glucose*, *salt or fat*, *or any of those things aren’t allowed*. *The biggest question is how to manage in life without eating anything*, *just vegetables all the time*. *And it’s hard because I am often outside the home’* (PWD, woman, FG-2).

Regarding physical activity, people with diabetes emphasized that fatigue (due to diabetes) and financial factors were barriers. One remarked: *‘If you engage in physical activity*, *even walking*, *it [glucose] decreases*. *I think it helps*, *but with the fatigue*, *it’s hard’* (PWD, man, FG-1).

Support from others, especially family, was raised as essential for achieving a healthy lifestyle (e.g., preparing healthy meals and encouraging physical activity). However, participants also found it challenging and annoying when others pressured them to eat unhealthy foods, especially in social meetings: *‘At parties it’s very hard*. *They know I’m diabetic and using insulin*. *It’s annoying when others pressure me to eat things that I should not’* (PWD, woman, FG-1).

**Hypoglycaemia** was described as a major problem. Participants shared their hypoglycaemic experiences and described the events: ‘*I felt like a* z*ombie; not connected to my surroundings*. *I almost lost consciousness many times*, *I had sweaty palms and felt severely weak’* (PWD, woman, FG-1).

Hypoglycaemic experiences were described as traumatic and participants feared experiencing them again: ‘*My biggest problem (…) was that for four months I had severe hypoglycaemia every night*. *I woke up in the middle of night looking for something to eat*, *I walked while sleeping*, *and I fell many times (…) I don’t want to be hypoglycaemic again’* (PWD, man, FG-1).

Experts highlighted the importance of asking individuals about their fear of hypoglycaemia: ‘*Fear of hypoglycaemic events is very important’* (Expert-E, diabetes nurse).

**Sexual dysfunction** due to diabetes was raised in the groups by both people with type 2 diabetes and experts. A PWD mentioned:

‘*Diabetes negatively influences many things*, *I was in bad condition*, *I was depressed*, *and it affected my social life*. *Let’s be honest*, *when a person has a high glucose level*, *their life with their partner changes*, *it’s not a normal life*. *Maybe the person won’t talk about all this*, *but when I lowered my glucose level*, *it gave [me] more power and more enjoyment’* (PWD, man, FG-2).

The experts particularly mentioned asking individuals about sexual dysfunction, which is relevant for both sexes. And, they also noted that this issue usually does not arise during medical visits. One diabetes physicians remarked: *‘I want to ask [using a questionnaire]*, *for example*, *about impotence*, *since we usually don’t ask*, *as it’s uncomfortable*, *since the patient is accompanied by a family member*. *And answering a questionnaire will be more comfortable for the patient’* (E, diabetes physician).

In addition to physical struggles, the **financial burden** associated with diabetes, primarily due to costs of medication, was evident from participants’ responses and appeared to be a barrier to treatment for some individuals. An elderly woman mentioned:

*‘There’s a new medication that’s effective*. *But the packet costs 250 shekels [73 USD]*. *What about the low-paid workers or the elderly who live off of their pensions*, *how could they pay for that*? *They cannot’* (PWD, woman, FG-2).

In addition, gym fees and the loss of productivity at work increased financial burden. A man remarked: ‘*You have to work out*, *it costs money*. *You’re less productive at work*, *that also costs money’* (PWD, man, FG-3). Additionally, a woman who experienced kidney failure said: ‘*I need to go to the gym*. *But the gym costs money*. *In our neighbourhood*, *there are no suitable parks for walking’* (PWD, woman, FG-2).

#### 2. Mental health issues

Diabetes is a demanding chronic disease, affecting physical and mental health. Individuals with diabetes experience a variety of emotions and mental health issues, including depression, distress, anxiety, fear, frustration, and loneliness. Mental health was one of the most prominent issues emphasized by individuals and experts. One expert remarked: ‘*The mental aspect is important*. *It’s important to ask not only about depression*, *but also fear*, *worry and anxiety’* (E, family physician). PWD suggested: ‘*I think they [health plans] should employ a health-provider*: *a physician or a nurse to address the individual’s psychological issues’* (PWD, man, FG-3).

A diabetes diagnosis requires that individuals implement lifestyle modifications and restrictions, which may cause depression. For example, a newly diagnosed PWD reported: *‘Diabetes causes depression because suddenly you have to change your lifestyle completely*. *You have to think about what you put in your mouth*, *what you do*, *you need to sleep well without getting up to go to the bathroom several times’* (PWD, man, FG-3).

Another cause of depression PWDs mentioned was needing medications, especially among those needing multiple medications.

*‘When going to the pharmacy*, *people feel as if they’ve been to the supermarket*. *You collapse immediately after carrying all the medications*. *You’ll be depressed just from the number of medications’* (PWD, man, FG-2).

Anxiety and fear over developing diabetes complications were the dominant mental health related aspects mentioned across all groups: *‘My father had diabetes and*, *at age 80*, *his leg was amputated*. *This led to many thoughts*, *to fear and anxiety’* (PWD, woman, FG-1). A newly diagnosed PWD related: ‘*I read about diabetes and its complications; it scares me’* (PWD, man, FG-3).

Preventing complications was considered the most valuable outcome among participants. As one PWD mentioned: *‘I want to die healthy*, *I don’t want all these complications*, *I don’t want to experience these complications’* (PWD, man, FG-3).

The experts mentioned that mental health aspects typically do not arise during medical appointments, suggesting that health providers could use PROMs as a signalling system to help assess when and with whom they should address mental health issues. An expert remarked: *‘We need to add some questions [PROMs]concerning diabetes complications*, *since this is what we’re trying to prevent’* (E, diabetes physician).

#### 3. Self-management ability

A meaningful domain was the capacity of self-management, i.e., the ability to manage the lifestyle modifications inherent to living with diabetes, having symptoms and managing treatment. The person with diabetes is the cornerstone of the treatment process, as a PWD emphasized: ‘*The treatment is in her own hands [the individual’s responsibility] and not in that of the physician’s (…) I was determined to reduce my glucose levels’* (PWD, man, FG-2). Diabetes physician mentioned: ‘*Lack of treatment empowerment is one of the problems*. *Individuals should advance the success of their treatment’* (E, social worker).

People with diabetes make daily decisions regarding food, activity and medications. To make the right decisions, individuals need guidance from their health providers. Individual empowerment, being informed about one’s disease and treatment, stood out as essential for successful self-management. However, individuals complained that they did not receive enough information from their health providers: *‘There’s a lack of information*. *I have no idea what to do*. *I would like to comprehend the information and not only to receive instructions*, *to understand what I’m doing’* (PWD, woman, FG-2).

Lack of knowledge about the disease, especially among the newly diagnosed, caused confusion. To prevent confusion, participants preferred receiving relevant information from health providers rather than searching on the internet.

‘*The problem is that*, *(…)*, *I just don’t know*! *There is diabetes type 1 and type 2*. *I’ve read a lot*, *but I don’t know what is relevant to me and what is not*. *(…) Since there is lack of knowledge*, *the more I read*, *the more I’m [another participant*: *more worried*, *more scared]*, *yes*, *more scared but also more confused’* (PWD, man, FG-3).

PWDs believed that raising awareness about diabetes and its treatment would increase compliance:*‘Today*, *patients have many sources of information to read about diabetes*. *I think it could be problematic*. *If we have an informational or support group*, *we can learn about diabetes*, *and its complications*. *It could increase patient’s compliance* (PWD, woman, FG-1).

#### 4. Patient-clinician relationship

The patient-clinician relationship was prominent in all the discussions and primarily reflected participant demands.

*The* ***devotion*** of clinicians was emphasized by the PWDs as a critical factor increasing their adherence: *‘I go to my check-up by him [the physician] after some time has passed and I know he’s devoted so much to my care*. *He wants to help me and then*, *what*? *I won’t listen to him*? *No*, *he’s helped me so much’* (PWD, man, FG-1).

***Trust in*** clinicians and the treatment is a key component of the treatment process according to the experts. One diabetes physicians stated: ‘*A very simple question I ask myself with every patient is*, *does the patient believe in me*? *Does the patient believe in the medications I give him*? *These are the two main questions’* (E, diabetes physician).

Another physician remarked: ‘*The key question is*: *Does the patient believe in his treatment*? *It is highly important for diabetes care*. *It may not be so important for other diseases*, *such as cancer and multiple sclerosis*, *for which patients believe completely in their treatment*. *In diabetes*, *there is much less belief and patients want natural treatment’* (E, diabetes physician).

Indeed, PWDs stated that they do not trust antidiabetic medications and believe they are harmful and cause complications. As one PWD noted: *‘The pills are harmful*, *40% of patients have kidney disease complications because of medications’* (PWD, man, FG-2). In another group, a newly diagnosed PWD reported: *‘I have read about medications that cause harm*. *Yes*, *they’re harmful in the long term*. *Maybe not in the short term*, *but if you take medications for years it’s not good’* (PWD, man, FG-3). One PWD summarized: ‘*I think we need someone to guide patients who don’t want to take medications*, *maybe to go in a natural way*, *use alternative medicine*. *Why can’t the health* plan*s suggest a solution for this*? *Or maybe they do*, *but we don’t know about it’* (PWD, woman, FG-1).

***Shared decision-making*** was another demand that people with diabetes raised. They wanted an informative treatment plan and the option to choose another treatment plan if they do not accept the proposed treatment, medications, or diet.

‘*I don’t want to be given a limited approach*. *What did the dietitian tell me*? *This is the diet that I give you and if you don’t (…) [accept]*, *I have nothing more to say*. *Also*, *the physician said*, *Why are you coming to me if you don’t agree with the treatment*?. *There should also be an alternative (…) they should tell me*, *okay*, *there is A and there is B*. *There should be more than one option but that does not exist’* (PWD, man, FG-3).

***Multidisciplinary care*** under one roof and during the same visit was a highly important factor among individuals with diabetes. They indicated that not having this could be a treatment barrier.

‘*When it’s all under one roof and provided at the same time*, *it’s much more efficient (…) it’s easier*. *It’s not that today I’m going to a family physician or a diabetes physician and on Thursday I have to go to a dietitian’* (PWD, woman, FG-1).

Expert discussions revealed that health providers favour using PROMs in diabetes care and even acknowledged their necessity to improve the treatment process, particularly regarding sensitive issues such as mental state and sexual dysfunction. One of the experts summarized: ‘*I think these measures [PROMs] are more important than some measures that we have today*. *I say this definitively*, *I would like to see more of these measures and less of the clinical measures’* (E, quality of care expert). However, many of the sub-domains that were raised by the people with type 2 diabetes were not mentioned in the experts’ panels. Table 1 presents the similarities and differences between perspectives of people with type 2 diabetes and healthcare providers. All the sub-domains that were raised by the healthcare providers were raised also by the people with type 2 diabetes.

**Table 1:** Valuable aspects for type 2 diabetes; perspectives of people with type 2 diabetes and healthcare providers.

Sub-domains that were raised only by people with type 2 diabetes	Sub-domains that were raised by both; people with type 2 diabetes and healthcare providers
• Physical function and fatigue	• Hypoglycaemia
• Healthy lifestyle struggles	• Sexual dysfunction
• Financial burden	• Mental health issues
• Lack of knowledge about the disease	• Self-management ability
• Devotion of clinicians	• Trust in clinicians & the treatment
• Shared decision-making	
• Multidisciplinary care in the same visit	

## Discussion

This study identified valuable aspects for people with type 2 diabetes that can be used as a basis for the development of PROMs in diabetes care. The analyses revealed four domains. First, several challenges that people with type 2 diabetes reported facing included decreased physical function, fatigue, hypoglycaemia, struggles with implementing a healthy lifestyle, sexual dysfunction, and financial burdens. Second, various negative emotions and mental health issues accompanied life with diabetes, including anxiety, distress, loneliness and depression. Third, self-management ability arose as a cornerstone of the treatment process which could be improved with guidance and support by healthcare providers. Fourth, the quality of the patient-clinician relationship was emphasized, including the importance of shared decision-making, and trust in their treating clinicians and prescribed antidiabetic medications. Our results suggest that PROMs based on perspectives of people with type 2 diabetes could address the gaps between patients’ perspectives and the healthcare providers’ perspectives, and promote patient-centered care.

Our findings indicate that challenges of people with type 2 diabetes (reduced physical function, hypoglycaemia, and struggles with implementing a healthy lifestyle) interrupted their daily activities and lowered individuals’ general health. This is in line with previous studies [20, 29–31]. A recent review of PROMs tools for diabetes found that the most common tools are related to quality of life [13]. Tools that exist in the literature include generic questionnaires, such as Patient-Reported Outcomes Measurement Information System (PROMIS-10) [32], while others are diabetes-specific, such as Audit of diabetes-dependent quality of life (ADDQoL) [33]. Diabetes is associated with many comorbidities and complications, and it is difficult for people with type 2 diabetes to discern the effects of diabetes and its treatment (e.g. fatigue) from diabetes complications and from other comorbidities, therefore, a generic instrument may be more suitable than a diabetes-specific tool for the general health assessment.

Our study identified sexual dysfunction as a topic that is usually not discussed during medical appointments, and PROMs could be helpful in addressing this sensitive issue. Up to 85% of people with type 2 diabetes have some level of sexual dysfunction [34], which is a central concern for both men and women [35]. However, the problem is often neglected in clinical practice as people expect their health providers to initiate this discussion, whereas practitioners usually fail to do so [35]. Recent review papers focused on PROMs in diabetes and described the dimensions that have been measured in the literature [13, 14, 36], but sexual dysfunction is often neglected also in current PROMs tools. Our study emphasizes the importance of addressing this neglected issue using PROMs.

Although Israeli citizens receive universal health coverage, the treatment of diabetes impose financial burden due to medication costs, gym fees and loss of work productivity. This is in agreement with a recent survey we analysed, where Israeli people with diabetes complained on the financial burden of medications (unpublished data, manuscript in preparation). A previous study in Israel found that 10% of people with diabetes are non-adherent with medications due to cost [37]. In the United Sates, studies have shown that people with diabetes face a financial burden related to their diabetes [38, 39]. Nevertheless, there is no common consensus about PROMs tools to measure financial burden among people with type 2 diabetes [13, 14, 21, 36].

The results show that diabetes has a major effect on individuals’ mental health. Consistent with our results, previous studies have shown that diabetes is associated with a wide range of emotional conditions, including stress, anxiety, fear, frustration, loneliness, guilt and depression [38–40]. The term ‘diabetes distress’ commonly refers to the wide range of emotional states that individuals with diabetes experience [41]. It is highly prevalent among adults, has negative impacts on self-care behaviors [41] and is recommended for routine monitoring in diabetes care [10, 19]. Several tools were developed to measure diabetes distress and the most commonly used tool is Problem Areas in Diabetes (PAID) [13]. Recently, the International Consortium for Health Outcomes Measurement (ICHOM) recommended using PAID as the standardized diabetes-specific tool for PROMs [19].

This study found that self-management ability is a valuable aspect for people with type 2 diabetes, and healthcare providers have a crucial role in enhancing people’s abilities to manage their diabetes by supplying information about the disease and its treatment. However, people with type 2 diabetes in this study and prior studies conducted in other countries [20, 42, 43] noted that they did not receive sufficient information from clinicians, or did not understand the provided information. Our findings, in line with previous study [44], indicated that the patient-clinician relationship is an important aspect for people with diabetes, affecting self-care behaviours and treatment adherence. It is recommended that physicians provide people with more information and engage them in shared decision-making to enhance individuals’ trust [45].

Our study showed that there are content gaps between perspectives of people with type 2 diabetes and those who treat them, which is one of the main contributions of this study. For example, topics such as the financial burden related to diabetes management (treatment and healthy lifestyle), and the importance of shared decision-making were raised by people with type 2 diabetes but not by healthcare providers, and thus might be missed in provider-influenced PROMs. Notably, many tools were developed on the basis of the experts’ perspectives [46]. In addition, our study emphasized important yet sensitive topics, such as sexual dysfunction, which are somewhat neglected in the existing PROMs. A recent systematic review found that people with type 2 diabetes and their healthcare providers differ in their perspectives on quality of diabetes care, and the authors argued that including perspectives of both groups in the redesign of type 2 diabetes care can help in overcoming challenges of diabetes care [47].

Our study had some limitations. First, data saturation had been reached with relatively small number (n = 19) of people with type 2 diabetes. Indeed, a recent review confirmed that qualitative study can reach saturation at relatively small sample size [25]. The small sample size could be related to the homogenous characteristics of the Israeli healthcare framework in which all residents have health coverage, that includes a standard, predetermined, basket of medical services, and the quality of diabetes care is routinely measured on a national level. Second, the study focused on type 2 diabetes (accounting for 90% of all diabetes cases [48]); thus, meaningful issues specific to type 1 diabetes were not investigated. Third, FGs of people with type 2 diabetes were led by the same researcher. However, credibility was strengthened by different perspectives being represented in the research group [49]. Fourth, the study was based on data from 2017–18, it maybe that current aspects of people with type 2 are somewhat different but we believe that most elements are still relevant.

Our study also had some important strengths. First, it was based on the voices of people with type 2 diabetes. Various tools were used in the literature to assess PROMs in diabetes, but it is necessary to identify the needs and perspectives of people living with type 2 diabetes to choose appropriate tools from the existing validated tools and to add items if needed. Second, recruitment efforts were aimed toward participants with diverse characteristics, strengthening our study’s credibility [49]. Third, we added experts’ perceptions to provide triangulation of information and to assess whether experts were in favour of PROMs for diabetes routine care.

In conclusion, this study has identified four domains that are valuable for people with type 2 diabetes in Israel. These domains could be relevant to other countries and we recommend that researchers and healthcare providers intending to utilize PROMs for routine diabetes care consider the aforementioned domains. There are content gaps between perspectives of people with type 2 diabetes and their healthcare providers. PROMs are essential for people with type 2 diabetes and healthcare providers in addressing issues largely not addressed in routine diabetes care and were missed in provider-influenced PROMs. This study representing an important first step in the future routine use of PROMs in Israel. In future research regarding incorporating PROMs into diabetes care, we plan to assess associations between socio-demographic variables and clinical quality measures using PROMs.

## Supporting information

S1 File Semi-structured open-ended topic guide. (DOCX)
