# Supplementary material for: What is important for people with type 2 diabetes? A focus group study to identify relevant aspects for Patient-Reported Outcome Measures in diabetes care
Source: PLoS One. 2022 Nov 14;17(11):e0277424. doi: 10.1371/journal.pone.0277424 (PMC9662717; doi:10.1371/journal.pone.0277424)
Supplement: S1 File — (DOCX) [file pone.0277424.s001.docx]

**What is important to people with type 2 diabetes?
A focus group study to identify relevant aspects for Patient-Reported Outcome Measures in diabetes care**

**Supplementary file** Semi-structured open-ended topic guide

- Introduction to study and objectives
- Ethical issues
- Demographic questionnaire

1. Can you describe your experience living with diabetes?

(Probe: your daily life, mobility, career, social relationships)

1. Can you describe how diabetes affects you emotionally and physically?

(Probe: barriers and challenges to live a full life with diabetes)

1. What can help you to live a full life with diabetes?
2. What feelings do you have when thinking about diabetes?

(Probe: nothing special, nervousness, fear, frustration)
 - If fear/frustration are mentioned: (afraid of ? frustrated about?)

1. Please tell us about the diabetes treatment you receive.
   (Probe: your medical appointments, opinion on diabetes care, experience in the clinics)
2. What are your expectations from diabetes treatment?

(Probe: what is important to achieve? what is important to avoid?)

1. How could you measure the quality of diabetes care that you have received

Probe:
 The treatment is good since it helps me with…
 The treatment is not good since it does not help me with…
